# Thermostabilization of a fungal laccase by entrapment in enzymatically synthesized levan nanoparticles

**DOI:** 10.1371/journal.pone.0304242

**Published:** 2024-07-18

**Authors:** Hossein Alishah Aratboni, Maura Martinez, Clarita Olvera, Marcela Ayala

**Affiliations:** Departamento de Ingeniería Celular y Biocatálisis, Instituto de Biotecnología UNAM, Cuernavaca, Morelos, Mexico; Banaras Hindu University, Varanasi, UNITED STATES

## Abstract

In this work, we present a comprehensive investigation of the entrapment of laccase, a biotechnologically relevant enzyme, into levan-based nanoparticles (LNPs). The entrapment of laccase was achieved concomitantly with the synthesis of LNP, catalyzed by a truncated version of a levansucrase from *Leuconostoc mesenteroides*. The study aimed to obtain a biocompatible nanomaterial, able to entrap functional laccase, and characterize its physicochemical, kinetic and thermal stability properties. The experimental findings demonstrated that a colloidal stable solution of spherically shaped LNP, with an average diameter of 68 nm, was obtained. An uniform particle size distribution was observed, according to the polydispersity index determined by DLS. When the LNPs synthesis was performed in the presence of laccase, biocatalytically active nanoparticles with a 1.25-fold larger diameter (85 nm) were obtained, and a maximum load of 243 μg laccase per g of nanoparticle was achieved. The catalytic efficiency was 972 and 103 (μM·min)^-1^, respectively, for free and entrapped laccase. A decrease in k_cat_ values (from 7050 min^-1^ to 1823 min^-^1) and an increase in apparent Km (from 7.25 μM to 17.73 μM) was observed for entrapped laccase, compared to the free enzyme. The entrapped laccase exhibited improved thermal stability, retaining 40% activity after 1 h-incubation at 70°C, compared to complete inactivation of free laccase under the same conditions, thereby highlighting the potential of LNPs in preserving enzyme activity under elevated temperatures. The outcomes of this investigation significantly contribute to the field of nanobiotechnology by expanding the applications of laccase and presenting an innovative strategy for enhancing enzyme stability through the utilization of fructan-based nanoparticle entrapments.

## Introduction

Laccases (benzenediol oxygen reductases, EC 1.10.3.2) are a family of copper-containing polyphenol oxidases that belongs to the multicopper oxidases [1]. Due to their low substrate specificity, laccases are able to catalyze the one-electron oxidation of a wide range of organic compounds, including phenols and aromatic amines, with the simultaneous reduction of molecular oxygen (O_2_) to water (H_2_O) [2, 3]. The catalytic reaction of laccase involves the removal of one electron from the substrates for the production of free radicals. The free radical is then subjected to homo- and hetero-coupling in order to form a dimeric product, a polymeric product, or a cross-coupling product, which has practical implications for organic synthesis [4]. Therefore, laccases are eco-friendly and versatile enzymes that exhibit significant potential in the synthesis of bioactive compounds, offering applications in the fields of medicine, pharmaceuticals, agriculture, producing cosmetics, nanobiotechnological productions, textile industries, woodworking industries, and food industries [5, 6]. Aside from its long-studied use in bioremediation and lignin degradation, other high-value applications for laccase have been described, for example the synthesis of compounds with potential medical use [7]; the synthesis of insulating polymers [8]; and as part of biosensors for pollutants, drugs and even for detecting infections [9–11]. For some of these possible uses of laccase, its combination with nanomaterials has been proven beneficial in terms of its activity, stability and suitability for different applications [12–14]. One significant culprit of mesophilic enzymes is the inherent limitations due to its lack of robustness [15]. Enzymes may exhibit vulnerability towards high temperature, extreme pH, the presence of organic solvents, oxidation, and shear stress due to mechanical agitation, consequently leading to diminished operational stability under certain conditions [16]. The immobilization of enzymes to preserve their activity under harsh conditions, and also to recycle them, has been widely studied for decades. Technological advances have allowed the synthesis, characterization and application of nanomaterials in several fields. Entrapment of bioactive molecules into nanoparticles enhances their stability, enables controlled release, and facilitates their efficient utilization across a broad spectrum of disciplines. As a result, this technology holds immense potential for transformative advancements in the realms of medicine, agriculture, environmental science, and beyond. In the particular case of laccases, several works demonstrating the amenability of these enzymes for immobilization on different types of supports, as well as their advantages and disadvantages, have been recently reviewed by numerous authors [10, 17–20].

In this research work, we have successfully coupled the synthesis of levan nanoparticles (LNP) with laccase entrapment. Levan is a biocompatible natural polymer produced by plants and bacteria through the action of levansucrases [21]. It is comprised of fructose monomers, covalently linked through β-(2→6) glycosidic bonds, with β-(2→1) linkages that introduce branching points within the polysaccharide chain [22, 23]. Levan is usually a high molecular weight polymer (ranging from 10^5^−10^9^ Da) with multiple branching (as high as 20%) [24]. This and other polysaccharides have been shown to form nanostructures either spontaneously during its enzymatic synthesis [25, 26] or under the influence of external agents [27–29]. Levan has been shown to entrap small molecules and proteins, and thus could also serve as nanocarrier for some applications [30, 31]. By utilizing these LNPs, we aimed to enhance the stability of a fungal laccase, thereby expanding its potential applications in various fields, including biocatalysis, biomedicine, and biosensing (for a recent review of enzyme immobilization into polysaccharides, see Sharma et al 2021 [32]; for a recent review on the advantages and applications of levan, see Domżał-Kędzia et al 2023 [31].

## Materials and methods

### Chemical reagents and enzyme

The chemicals employed in this study were sourced exclusively from analytical grade materials. The 3,5-dinitrosalicylic acid (DNS) was purchased from Thermo Fisher Scientific Inc. Syringaldazine, succinic acid and calcium chloride were purchased from Sigma. The production and purification of laccase from *Coriolopsis gallica* UAMH 8260 was conducted following previously established protocols, as documented in prior literature [33].

### Expression of the recombinant levansucrase (LevS△N70Tn38)

The pET-22b-LevS△N70Tn38 vector, containing a truncated version of levansucrase from *Leuconostoc mesenteroides* B512F [34] was introduced into electrocompetent *E*. *coli* BL21 cells obtained from New England Biolabs. The preparation and transformation of electrocompetent cells was performed according to the protocol provided by MicroPulser™ Electroporation Apparatus Operating Instructions and Applications Guide (Catalog Number 165–2100) Bio-Rad. The vector contained an ampicillin resistance gene, enabling the selection of positive clones, along with an origin of replication and a LacI promoter that induces transcription of the inserted gene. To initiate the transformation process, bacterial cells were cultured in 1 ml of Luria-Bertani (LB) medium and incubated at 37°C with continuous shaking at 350 rpm for 1 hour. This step aimed to allow the cells to reach the logarithmic growth phase, ensuring optimal conditions for the subsequent transformation procedure. Following the recovery period, the transformed cell suspension was evenly spread onto LB agar plates supplemented with 200 μg/mL of ampicillin. The plates were subsequently incubated at 37°C for 14 h.

Expression of LevS△N70Tn38 was performed as previously reported [25]. Transformed colonies were cultivated in Luria Bertani broth supplemented with 200 μg/mL ampicillin. The culture was incubated at 37°C and 200 rpm until reaching an optical density between 0.5 to 0.6 (measured at OD600, λ = 600 nm). At this point, induction of the culture was initiated by adding 1.5 mM isopropyl β-D-1-thiogalactopyranoside (IPTG). The induced culture was further incubated for an additional 8 h at 18°C and 120 rpm. After completion of the incubation period, the cells were harvested through centrifugation at 4°C, 9000 rpm for 10 minutes. The resulting cell pellet was subjected to two washes with a 10 mM phosphate buffer (pH 6.0). Subsequently, the washed pellet was resuspended in 20 mL of a 1 mg/mL lysozyme solution prepared in a 10 mM phosphate buffer (pH 6.0). Following a 30-minute incubation on ice for cell lysis, the samples underwent three cycles of freezing and thawing. Sonication was performed immediately using a 2-minute cycle at 70% amplitude, with intervals of 10 seconds on and 30 seconds off. Cell debris was subsequently removed by centrifugation until clarification of the extract was achieved (45 min at 9000 rpm and 4°C). The resulting supernatant containing the enzyme was collected and utilized for purification.

### Purification of LevS△N70Tn38

The LevS△N70Tn38 enzyme was subjected to purification through anion-exchange chromatography with Macro-Prep DEAE resin (Bio-Rad). The purification process involved continuous elution at a flow rate of 1 ml/min using an external peristaltic pump (Econo-Pump; Bio-Rad). The elution was performed with phosphate buffer at pH 6.0, employing a gradient ranging from 0.1 M to 1.0 M. Fractions demonstrating significant enzymatic activity, as assessed through the 3,5-dinitrosalicylic acid (DNS) assay, were pooled together and concentrated employing a stirred ultrafiltration cell (AMICON, Millipore, Darmstadt, Germany) with 10-kDa molecular weight cut-off membrane. The ultrafiltration process enabled the removal of smaller molecules and impurities, while retaining the desired LevS△N70Tn38 enzyme within the retentate.

### Quantification of levansucrase activity through a reducing-sugars assay

LevS△N70Tn38 activity was determined by quantification of released reducing sugars from sucrose employing the DNS assay described by Miller in 1959 [35]. The enzymatic reaction was conducted in a reaction volume of 600 μL, containing 292 mM sucrose, 50 mM acetate buffer (pH 6.0), and 1 mM CaCl_2_. The reaction mixture was maintained at a constant temperature of 30°C with continuous stirring at 350 rpm. To measure the release of reducing sugars, samples were taken at specific time intervals to measure the optical density (OD) at 540 nm using a Bio Spectrometer® UV-Visible Spectroscopy System (Eppendorf, Germany). The OD was converted to reducing sugar concentration using a standard curve. The LevS△N70Tn38 activity was expressed as enzymatic units (U), defined as the amount of enzyme required to generate 1 μmol of reducing sugars per minute under the specified assay conditions. All determinations were performed at least in triplicate.

### Quantification of laccase activity through the syringaldazine assay

Laccase activity was quantified utilizing the syringaldazine assay, a colorimetric technique. based on monitoring the increase in absorbance at 530 nm, indicative of product formation. The assays were conducted in succinate buffer (60 mM, pH 4.5) with 0.05 mM of syringadazine. The slope of A_530nm_ vs time was obtained and converted to values of U/mL using ε = 64,000 M^-1^ cm^-1^ [36]. One enzymatic unit (U) is defined as the amount of laccase required to generate 1 μmol of the product per minute, under the specified assay conditions. The rate measurements were performed at least in triplicate using the Agilent 8453 UV-Visible Spectroscopy System (Santa Clara, CA, USA).

### Synthesis and preparation of LNPs

The synthesis of LNPs was performed in a 600 μl reaction volume, under optimal conditions for LNP formation. The reaction mixture contained sucrose at 200 g/L in a pH 6.0 50 mM acetate buffer supplemented with 1 mM CaCl_2_. The reaction was initiated by adding 2 U/ml of LevS△N70Tn38 and it was incubated at 30°C with constant stirring for 12 h. Laccase entrapment was tested by adding different amounts of enzyme, specifically 13, 33, 66, 99, and 132 μg of laccase, and each of these conditions is referred to as LNP-Lac 13 μg– 132 μg. After 12 hr, free, non-entrapped laccase was separated from LNPs by employing an ultracentrifuge filter device with a 100-kDa molecular weight cut-off membrane (manufactured by Millipore, USA). The separation process involved centrifugation at a speed of 10,000 rpm for a duration of 10 minutes. Subsequently, the laccase-containing LNPs (LNP-Lac) retained in the filter were subjected to two washes using a 50 mM pH 6.0 acetate buffer. Following the washes, the LNPs were stored at a temperature of -20°C until further utilization and subsequent analysis.

### Quantification of laccase entrapment efficiency (EE) and load into the LNPs

The determination of laccase entrapment within the LNPs involved an indirect calculation method. This approach relied on quantification of the activity of free, non-entrapped laccase that was separated from LNP-Lac using an ultracentrifuge filter device, as described above. The amount of free laccase, obtained in a falcon tube, was quantified as enzymatic units (U) using the syringaldazine method. It is important to note that all reported values represent the average results obtained from three independent experimental replicates. Controls were performed to assess the efficiency of this procedure, by employing solutions of free laccase at different concentrations, performing the ultracentrifugation and measuring both retained activity after two washes, as well as recovered activity in the filtrate. Recovery efficiency of free laccase ranged from 68–70%, thus laccase entrapment quantification was corrected by a factor of 0.68. Data is available as supporting information, in S1-S3 Tables in S1 File.

The entrapment efficiency (EE) of laccase was determined using the following equation:

$$\%\text{EE}=\frac{IL-\left(\frac{FL}{0.68}\right)}{IL}\times 100$$

where IL represents the initial amount of laccase added to the LNP synthesis reactions, and FL is the amount of free laccase detected in the collected washes after the centrifugation process, and 0.68 is the correction factor.

### Effect of the presence of laccase on synthesis of levan

To assess the effect of laccase in the levan synthesis reaction, the produced polymer was quantified following the entrapment reactions. The levan polymer was recovered from the entrapment reaction by precipitation with ethanol; a 200 μL sample of the entrapment reaction was mixed with 300 μL of ethanol and incubated at 4°C for 1 h. After incubation, the mixture was subjected to centrifugation (15000 rpm, 20 minutes, 4°C), and the supernatant was completely removed. The resulting pellet was washed twice with 800 μL of absolute ethanol, followed by complete ethanol evaporation through incubation at 60°C for 10 minutes. Subsequently, the polymer was dissolved in 200 μL of 0.617 M HCl and subjected to hydrolysis at 80°C with constant stirring (450 rpm) for a duration of 2.20 h. The hydrolysis process was halted by adding 200 μL of 1 M NaOH, and the resulting polymer was quantified using the DNS method.

### Characterization of LNPs

For the characterization of the shape and morphology of the LNPs, transmission electron microscopy (TEM) was utilized. The TEM analysis was performed using a ZEISS model Libra transmission electron microscope, operating at an accelerating voltage of 120 kV. The LNPs samples were loaded onto a carbon-coated copper grid and subsequently air-dried prior to imaging. A Gatan digital camera was employed to capture high-resolution images for the comprehensive examination and characterization of the LNPs’ shape and morphology. To ascertain the polydispersity index (PDI), hydrodynamic size, and particle size distribution profile of the LNPs, dynamic light scattering (DLS) analysis was employed. The measurements were conducted using a Zetasizer NanoZS instrument (manufactured by Malvern Instruments Ltd., Malvern, UK). The DLS technique enabled the determination of the PDI, which indicates the degree of size variation within the LNP sample. Additionally, the hydrodynamic size of the LNPs was assessed, providing insights into their overall dimensions in a liquid medium. The Zetasizer NanoZS instrument facilitated the estimation of the particle size distribution profile, enabling a comprehensive understanding of the size range and distribution of the LNPs under investigation. To determine the surface charge of the LNPs, the zeta potential was measured using laser Doppler velocimetry (LDV) with the same instrument and experimental conditions. The zeta potential provides crucial information about the NPs’ surface properties, electrostatic stability, and colloidal behavior.

### Thermal and storage stability of entrapped laccase

A sample of LNP-Lac, as well as free laccase, was incubated for 1 h at 40, 50, 60, and 70°C. Enzyme activity measurements were conducted at 10-minute intervals, using the syringaldazine assay described above, to monitor any fluctuations or changes in activity levels. The residual activity of laccase was calculated by comparing the enzyme activity measured after 1 h-incubation at various temperatures (40, 50, 60, and 70°C) to the initial activity of the enzyme. The initial activity refers to the enzyme activity measured before the incubation period. The residual activity, expressed as a percentage, was calculated using the following formula:

$$Residual\;\text{Activity}(\%)=\frac{\text{Activity}\;\text{after}\;\text{incubation}}{\text{Initial}\;\text{activity}}\times 100$$

All assays were performed at least in triplicate.

The storage stability of LNP-Lac 33 μg, LNP-Lac 66 μg, and LNP-Lac 99 μg was evaluated over time by assessing their activity, through the syringaldazine assay, after 0, 6, 17, and 27 days of entrapment. The samples were kept at 4°C. Data is available as supporting information, in S4 and S5 Tables in S1 File.

### Kinetic characterization LNP-Lac

Different concentrations of syringaldazine (from 0 to 60 μM) were used to measure initial reaction rate of free and LNP-Lac 99 μg. All measurements were performed at least in triplicate. Data was fitted through non-linear regression to the Michaelis-Menten equation to obtain the values of Vmax and Km. For LNP-Lac, the amount of calculated entrapped laccase was used to obtain the k_cat_. Data is available as supporting information, in S6 Table in S1 File.

## Results and discussion

### Quantification of entrapment efficiency (EE) percentage

Recombinant LevS△N70Tn38 was produced from *E*.*coli* and purified to a specific activity of 350 U/mg with an electrophoretic purity of 90%. This enzyme was used to catalyze the production of levan, both in the absence and presence of laccase from *Coriolopsis gallica*. This laccase is a good model for high-redox fungal laccase, as it has been reported to be a very versatile catalyst, amenable to pilot-plant production, as well as protein engineering [33, 37, 38]. It is a glycosylated, monomeric protein with a molecular weight of 61 kDa and featuring typical properties of fungal laccases, such as an acidic pI of 3.4, an optimum pH between 4.5 and 5.5, and broad specificity towards numerous substrates [39–43].

**Fig 1:**
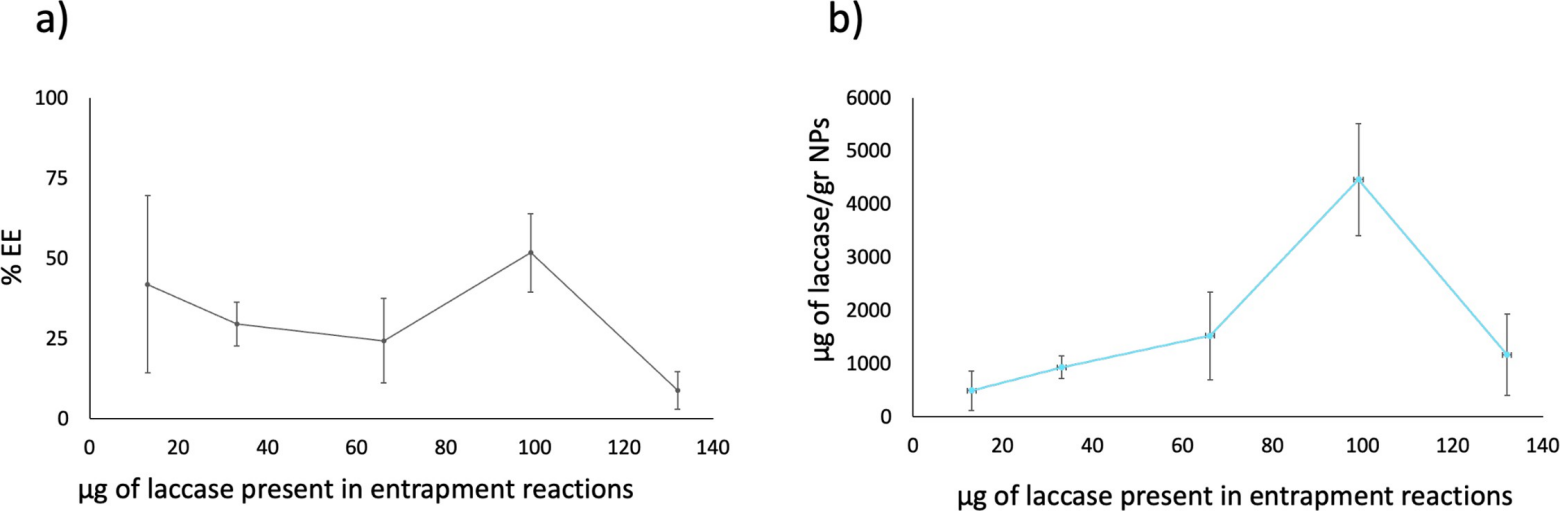
Comparative analysis of entrapment efficiency (EE) percentage for various amounts of laccase (a), and LNP laccase-load capability (b).

The efficiency of laccase entrapment within the LNPs was determined for different laccase concentrations, namely LNP-Lac 13 μg, LNP-Lac 33 μg, LNP-Lac 66 μg, LNP-Lac 99 μg, and LNP-Lac 132 μg. The EE were found to vary depending on the laccase concentration, reaching average values of 42, 30, 24, 52, and 9%, respectively (Fig 1A). Among the tested concentrations, LNP-Lac 99 μg exhibited the highest EE at 52%. In contrast, LNP-Lac 132 μg showed the lowest EE at 9%, suggesting a saturation effect that at high laccase concentration leads to a decrease in EE.

**Fig 2:**
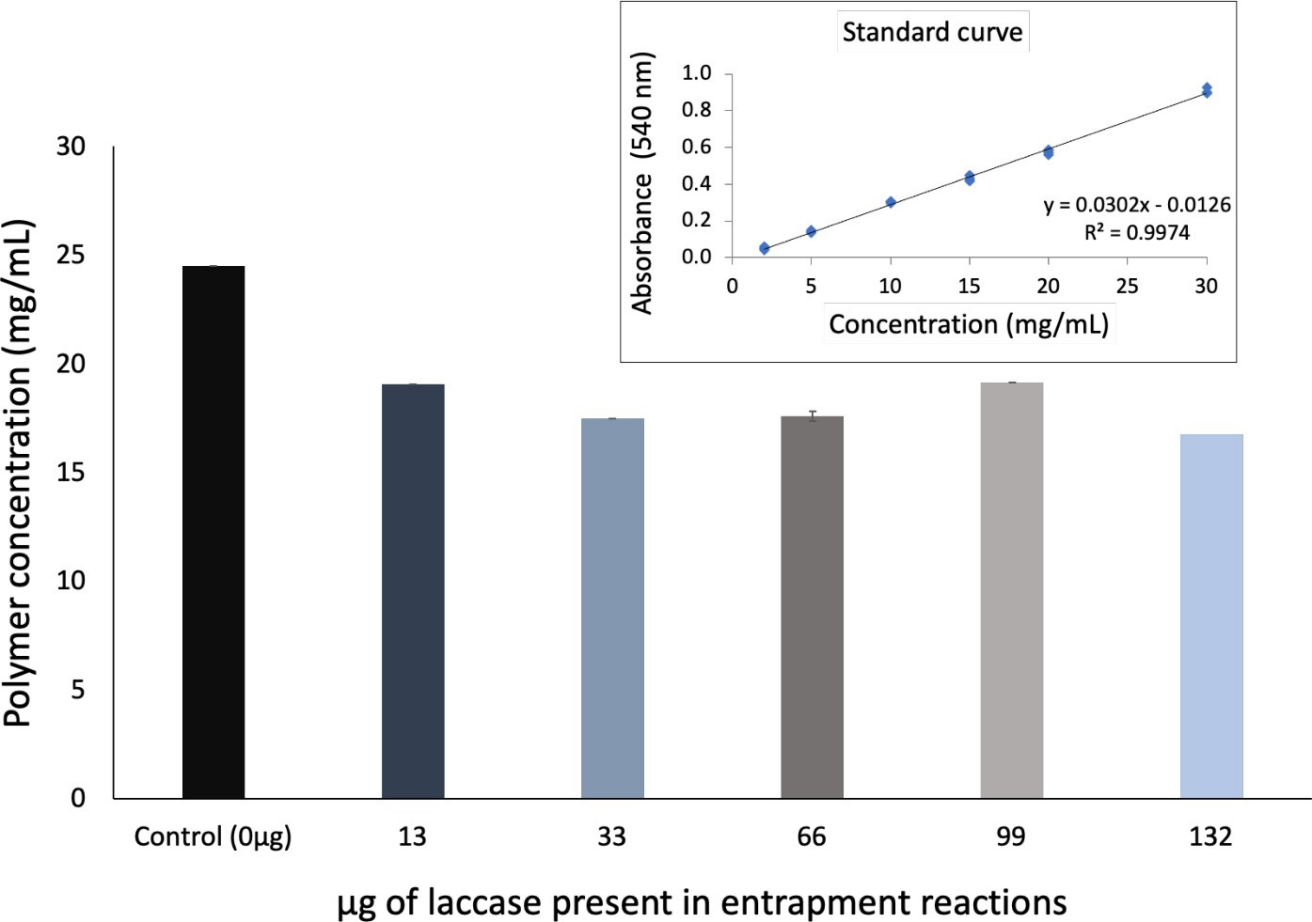
Quantification of levan produced by the LevS△N70Tn38-catalyzed polymerization reaction, in the presence of different amounts of laccase.

To assess the potential impact of laccase entrapment on the extent of levan production, the amount of synthesized polymer was quantified both in the absence and in the presence of laccase and is shown in Fig 2. The levan concentration was 24.5 mg/mL in the absence of laccase, and decreased to 19.1, 17.5 17.6, 19.1 and 16.7 mg/mL, respectively, in the presence of the different amounts of laccase used in entrapment reactions. Laccase does not interact or recognize sucrose, fructose or glucose as substrate or ligand, so a decrease of 20 to 35% in the amount of obtained levan cannot be explained in terms of competing/inhibiting secondary reactions. Nevertheless, the integrity and formation of catalytically active LNP were not compromised.

Regarding the laccase load (amount of laccase per gram of levan nanoparticle), it also provided valuable information about the efficiency of the entrapment process and can guide the optimization of enzyme loading for various applications. Therefore, following the determination of EE and the amount of produced levan, the amount of laccase (in μg) entrapped per gram of LNP was calculated. The results demonstrated varying laccase loading capacities. The amount of laccase that was entrapped per gram of LNP was found to be 496 μg, 931 μg, 1524 μg, 4468 μg, and 1166 μg, respectively (Fig 1B). These findings reveal a clear trend in laccase loading capacity with respect to the enzyme concentration utilized during the entrapment process. The highest laccase loading capacity of 4468 μg/gr NP was achieved when employing an amount of 99 μg of laccase in the entrapment reaction. However, a higher laccase amount of 132 μg resulted in a lower load of 1166 μg/gr LNPs, indicating again a saturation effect. The sample LNP-Lac 99 μg was used for further kinetic and stability characterization, as it was the best preparation in terms of laccase loading.

### Physical characterization of LNP and LNP-Lac

A comprehensive study of the LNPs physical properties, such as the average particle diameter, PDI, and surface charge was performed using DLS in combination with a zeta potential analyzer (Table 1). The average size was 68 nm for LNP and 85 nm for LNP-Lac 99 μg, respectively. These dimensions provide valuable information about the overall size of the nanoparticles, indicating their potential suitability for various applications. These values are similar to those reported by González-Garcinuño, *et al* in 2019 [26], who analyzed the size of LNPs synthesized by two systems: whole bacteria and cell-free system, producing nanoparticles of 90 nm and 110 nm respectively.

**Table 1:** Physical properties of LNPs and LNPs-Lac 99 μg.

Sample	Average particle diameter (nm)	PDI	Zeta potential
LNP	68	0.036	-0.64
LNP-Lac 99 μg	85	0.056	-1.92

Additionally, PDI values were calculated as 0.036 for LNP and 0.056 for LNP-Lac 99 μg. The PDI represents the degree of size distribution within the nanoparticle population, with lower values indicating a narrower and more uniform particle size distribution. The relatively low PDI values obtained for both LNP and LNP-Lac 99 μg suggest a relatively homogeneous nanoparticle formulation. Others works using the enzymatic method to produce fructan nanoparticles have reported PDI values lower than 1 [25]. The slight size and PDI differences between the LNPs vs LNP-Lac 99 μg, could be attributed to the entrapment of laccase as has been observed for other LNPs [27].

The zeta potential, serving as an indicator of the surface charge on nanoparticles, was -0.64 mV for the LNP and -1.92 mV for the LNP-Lac 99 μg samples. The slight negative value of zeta potential for laccase-containing LNP could be due to negatively charged laccase molecules, as the isoelectric point (pI) of this enzyme is 3.4 [38, 41]. Zeta potential plays a crucial role in determining the stability and potential interactions of nanoparticles within their surrounding environment. According to previous research by Barhoum et al. [44], nanoparticles with zeta potential values ranging from -10 to 10 mV are generally considered to be neutral. In line with these findings, the zeta potential values obtained in this study for the LNP and LNP-Lac 99 μg support their classification as neutral nanoparticles. Other works with proteins entrapped into LNP report similar zeta potential values. As reported by Oktavia et al [45] entrapment of bovine serum albumin (BSA) and lysozyme in levan, yielded nanoparticles with a non-spherical (for BSA) and spherical (for lysozyme) shape, an average size of 65–100 and 210–280 nm and negative zeta potential values of -4.72 and -2.57 mV, respectively. In the context of drug delivery systems and other biomedical applications, nanoparticles possessing a neutral charge demonstrate several advantageous characteristics. Specifically, these particles exhibit an extended circulation time within the biological system [46], enhanced stability, and a reduced propensity for nonspecific interactions with biological components [47].

**Fig 3:**
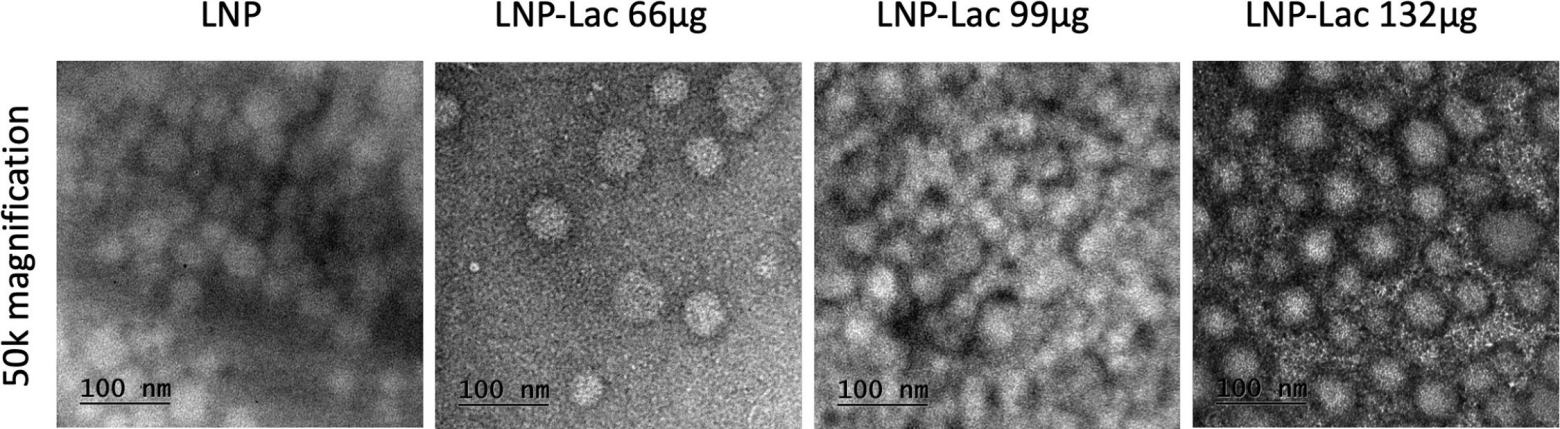
TEM micrographs of LNPs with different concentrations of laccase.

The analysis of images from TEM (Fig 3) revealed that the LNP, LNP-Lac 66 μg, lac 99 and LNP-Lac 132 μg samples exhibited predominantly spherical shapes with uniform sizes. These findings strongly suggest that the enzymatically synthesized levan possesses the inherent capability to self-assemble, leading to the formation of NPs. Similar reports on spontaneous nanostructuration are available [25, 26, 28, 48]. Most important, the entrapment of laccase during the enzymatic synthesis of levan is feasible. These results shed light on the potential of utilizing levan-based systems for NP formation and the incorporation of bioactive enzymes such as laccase. The successful demonstration of these principles in the present study holds promise for various applications in the field of nanotechnology.

The samples were subjected to examination on two separate occasions. The first evaluation by TEM took place immediately following the synthesis of the nanoparticles and the entrapment of laccase. The second evaluation occurred approximately one month after the entrapment, aiming to assess the long-term stability and condition of the nanoparticles. This follow-up analysis was carried out to ensure that the nanoparticles maintained their structural integrity and functional properties over an extended period. We observed the nanoparticles maintained its integrity after 27-day storage, as will be shown below.

### Kinetic characterization of LNP-Lac

The activity of LNP-Lac was measured with syringaldazine and for all cases, the biocatalyst displayed lower activity than expected on the basis of the amount of entrapped laccase (Table 2).

**Table 2:** Activity of different preparations of LNP-Lac.

Sample	Measured activity of LNP-Lac (U/g LNP)	Retained activity (%)
LNP-Lac 13 μg	12.4	42.4
LNP-Lac 33 μg	27.5	24.4
LNP-Lac 66 μg	54	26.3
LNP-Lac 99 μg	69.7	13.3
LNP-Lac 132 μg	99	63

**Fig 4:**
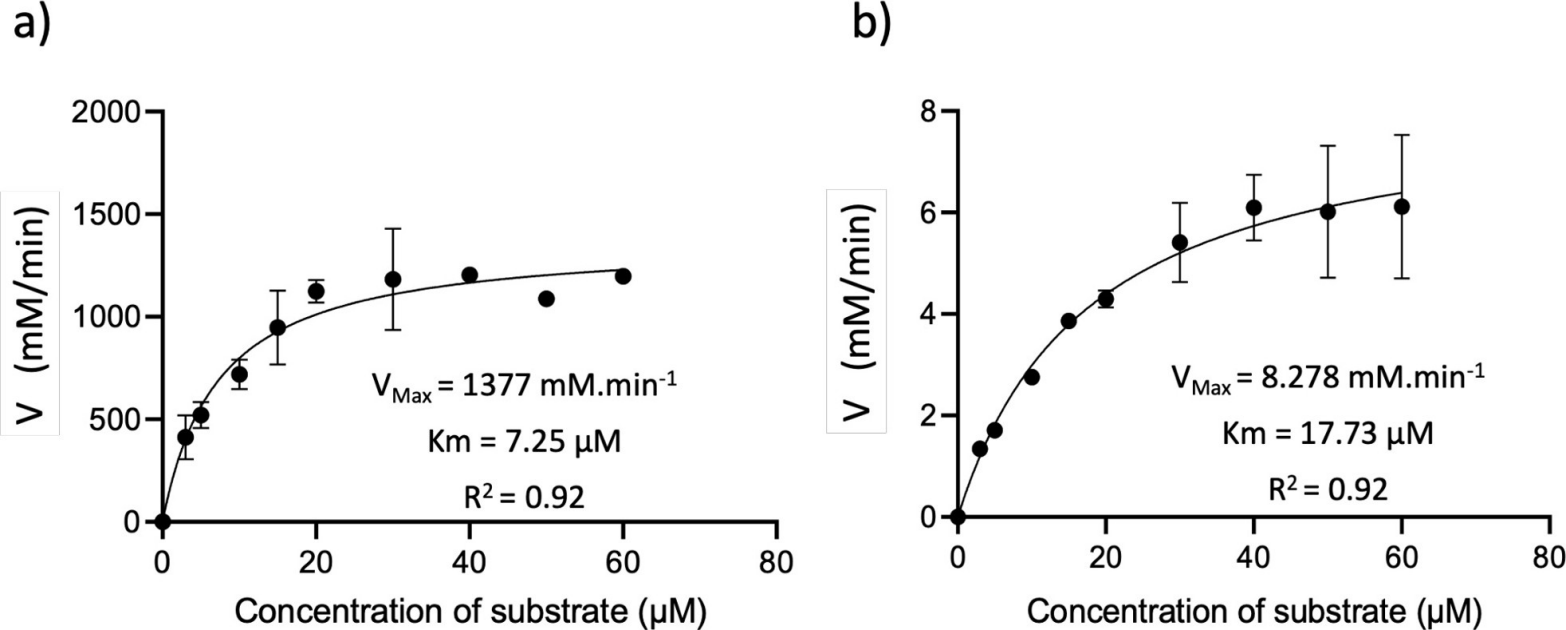
Determination of enzyme kinetics of (a) free laccase and (b) LNP-Lac 99 μg from data adjusted to the Michaelis-Menten model by non-linear regression.

In order to better understand this decrease in activity, the kinetic characterization was performed for both the free laccase and LNP-Lac 99 μg. The initial rates for syringaldazine oxidation at different substrate concentration was fitted to the Michaelis-Menten equation and the kinetic parameters Vmax and Km were obtained (Fig 4). The calculated k_cat_ values for the free laccase and LNP-Lac 99 μg were 7050 min^-1^ and 1823 min^-1^, respectively, whereas apparent Km is 2.4-fold larger for the entrapped laccase (17.73 μM vs 7.25 μM for the free laccase). The entrapment of laccase into the polysaccharide matrix may introduce structural changes in laccase, reduce its flexibility and/or affect the accessibility of the substrate to the enzyme’s active site, as has been observed in other works [10]. In this particular case, where no covalent interactions exist between the enzyme and the nanostructured polysaccharide, the structural effects may be low or absent, thus the activity decrease of the entrapped laccase could be mainly due mass transfer limitations. A lower catalytic activity is expected when enzymes are immobilized inside a matrix [16], and for laccases there are many examples reporting this phenomenon [49].

The catalytic efficiency, the kcat/Km value, was also calculated for both free laccase and LNP-Lac 99 μg, yielding values of 972 and 103 (μM·min)^-1^, respectively. This indicates a 9-fold decrease in the catalytic efficiency of entrapped laccase. This reduction falls within the range reported in other studies involving enzyme immobilization. For instance, Lloret et al. [50] observed a substantial decrease of 27-fold in the catalytic efficiency of laccase when entrapped in sol-gel matrices composed of semi-permeable polymers. Similarly, in a study conducted by Castro, A. et al., [51] the catalytic efficiency of laccase decreased by as much as 250-fold. Thus, the observed decline in catalytic efficiency upon immobilization aligns with the commonly documented phenomenon encountered during enzyme immobilization processes [52–54].

While entrapment offers advantages such as improved stability (see below), it also results in a reduction in the catalytic efficiency of laccase. The choice between the free laccase and entrapped laccase should consider the specific requirements of the application, balancing the benefits of entrapment with the desired catalytic performance. Further investigations using different substrates and varying experimental parameters would provide a more comprehensive understanding of the catalytic behavior of both the free laccase and LNP-Lac 99 μg.

### Thermal and storage stability of entrapped laccase

**Fig 5:**
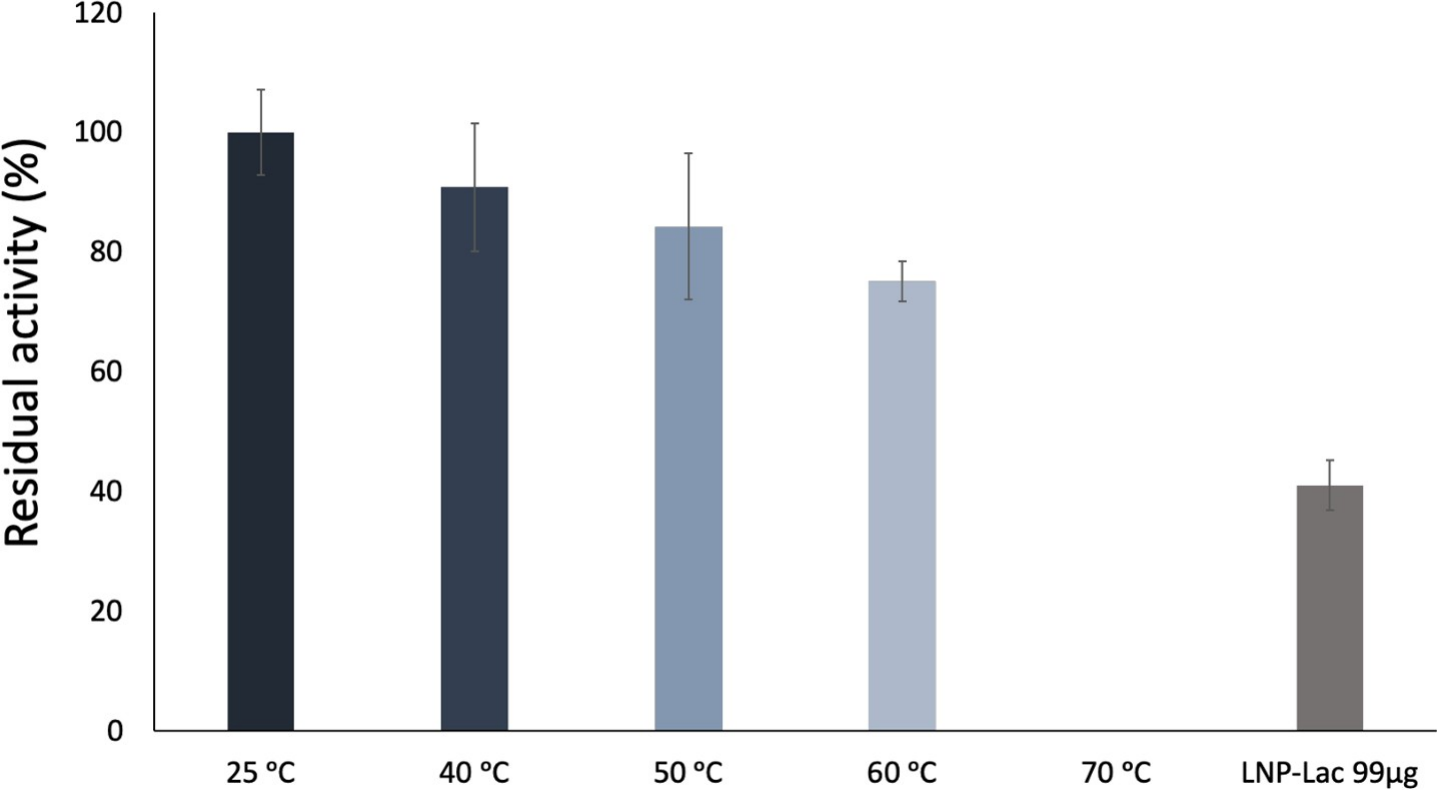
Effect of entrapment on laccase residual activity upon 1 h-incubation at various temperatures.

The temperature has a significant influence on enzyme activity, making the preservation of catalytic functionality essential for practical applications [55]. The environment provided by nanostructured hydrophilic macromolecules, such as levan, may be beneficial for maintaining the 3D structure of proteins, lowering their susceptibility to thermal inactivation. The thermal stability of free laccase was evaluated under various temperature conditions, including 40°C, 50°C, 60°C, and 70°C, over a duration of 60 minutes. Altough fungal laccases are robust enzymes due to glycosylation, and it maintains more than 75% after 1 h incubation at temperatures of 60°C and lower, we observed that the enzyme lost all activity after 1 h-incubation at 70°C (Fig 5). On the other hand, the entrapped laccase (LNP-Lac 99 μg) retained 41.01% residual activity under these conditions, indicating a remarkable improvement in thermal stability.

Comparing thermal stability of laccase with other works that also entrap it into natural polysaccharides, the environment within LNP appears to be more beneficial for retaining the laccase activity at high temperatures. Koyani et al, entrapped a laccase in chitosan NPs and reported no difference in activity loss for free laccase and entrapped laccase upon incubation at 60, 70, and 80°C over 30 min. The authors argue that chitosan NP seems to remain as a gel, thus unable to confer rigidity to the enzyme despite the electrostatic interaction that maintains the protein bound to the polymer [56]. In another investigation, laccase was entrapped into alginate beads [57]. The results showed that the immobilization did not increase the stability of the enzyme but instead led to a loss in the stability at high temperatures, together with enzyme leaching and degradation. In a different approach, in which a laccase was covalently immobilized into the surface of inulin-coated magnetic nanoparticles, approximately a 1.5-fold increase in thermal stability was conferred to the enzyme, as shown by residual activity after a 3 h-incubation at 75°C and pH ranging from 2.5 to 7.5 [58]. Inulin is also a fructan-based polymer, as levan. Apparently, fructan-mediated protection to thermal denaturation of enzymes cannot be generalized, as other proteins entrapped into LNP are only slightly stabilized; for instance, a xylanase entrapped into LNP showed a stability increase at 70°C of only 10%, based on the values of the inactivation constant for free and entrapped enzyme [59]. It is well known that polysaccharides exert a positive effect on the stability of proteins when exposed to harsh environments, for example high temperatures and low water content (drying) medium [60, 61]. The exact mechanism is however controversial. It has been proposed that the hydroxyl groups from the saccharide moieties may form hydrogen bonds, reducing local and global flexibility of the proteins, which translates in slower denaturation.

**Fig 6:**
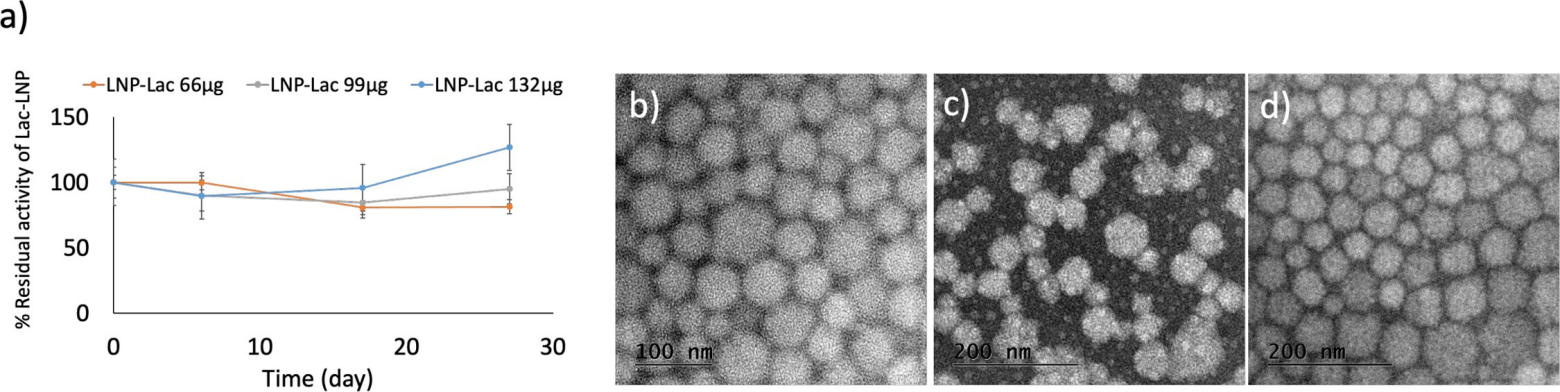
Time-course analysis of activity of LNP-Lac upon storage. (a) Residual activity of LNP-Lac 66 μg, LNP-Lac 99 μg, and LNP-Lac 132 μg at various storage times; TEM image of LNP-Lac 66 μg (b), LNP-Lac 99 μg (c), and LNP-Lac 132 μg (d) after 27 days-storage.

The storage stability of entrapped laccase into LNP was also determined, and provided valuable insights into the long-term stability and functionality of the entrapped enzyme (Fig 6A). As depicted in the figure, the activity of LNP-Lac 66 μg, LNP-Lac 99 μg and LNP-Lac 132 μg was assessed at 0, 6, 17, and 27 days after entrapment, resulting in high residual activity. The storage stability of the LNP-Lac suggests the robustness and stability of the entrapment system, allowing for the preservation of enzymatic functionality. The findings of this study in terms of improved stability are in line with that reported by other researchers for laccase [49, 62–64], which is actually one of the goals when immobilizing an enzyme. These results support the notion that enzyme entrapment into LNP can confer improved stability to the enzyme while maintaining a reasonable level of activity over an extended period.

Additionally, the structure and morphology of the nanoparticles were investigated to assess their stability after 27 days following the synthesis of laccase entrapment. Notably, it was observed that the structure and morphology of the nanoparticles were preserved, indicating the long-term stability of the entrapment system (Fig 6B–6D). The maintenance of the nanoparticle structure and morphology is crucial for ensuring the stability and functionality of the entrapped laccase. The results highlight the efficacy of the entrapment method in preserving the enzymatic performance of laccase, even during extended storage periods. The ability to maintain enzyme activity over time is crucial for practical applications, where stability is paramount for consistent and reliable performance.

## Conclusion

This study demonstrates the successful entrapment of laccase within fructan-based nanoparticles (LNPs), providing enhanced stability to the enzyme. The development of LNPs yielded stable and enzymatically active nanoparticles. The entrapped laccase within LNPs exhibited enhanced thermal stability, suggesting that the LNPs provided a protective environment for laccase, slowing down the temperature-induced denaturation. These findings may fuel the uses of laccase in medicine, pharmacy and biosensing. Immobilization of laccase into biocompatible polymers such as natural polysaccharides as levan, has the advantage to create biodegradable, non-toxic catalytic materials, to carry desirable enzyme activity to specific sites in which it is of use. For example, immobilized laccase into biocompatible polysaccharides could be applied as a component of wound dressings, to detect infections and in nanomedical devices [9, 37, 65]. Overall, this study contributes to the advancement of nanobiotechnology by demonstrating the successful entrapment of laccase within fructan-based nanoparticles resulting in an enzymatically active preparation that is also stable to temperature and long-term storage.

## Supporting information

S1 File File containing S1 to S6 Tables. (DOCX)
