# Supplementary material for: Thermostabilization of a fungal laccase by entrapment in enzymatically synthesized levan nanoparticles
Source: PLoS One. 2024 Jul 18;19(7):e0304242. doi: 10.1371/journal.pone.0304242 (PMC11257323; doi:10.1371/journal.pone.0304242)
Supplement: S1 File — (DOCX) [file pone.0304242.s001.docx]

| Volume of stock of laccase used for each entrapment reaction (µL) | Initial laccase activity in entrapment reaction (U) (equivalent in µg) | Name of sample |
| --- | --- | --- |
| 1 | 1.56 (13) | LNP-Lac 13 µg |
| 2.5 | 3.91 (33) | LNP-Lac 33 µg |
| 5 | 7.81 (66) | LNP-Lac 66 µg |
| 7.5 | 11.72 (99) | LNP-Lac 99 µg |
| 10 | 15.62 (132) | LNP-Lac 132 µg |

S1 table.  **Volume of concentrated laccase solution and the corresponding values of U and µg used in each entrapment reaction**. Initial units and µg were calculated according to the volume of a stock of laccase used in each entrapment reaction. The enzyme concentration and volumetric activity were 13.24 mg/mL and 1562 U/mL, respectively.

| Initial laccase activity in entrapment reaction (U) (equivalent in µg) | Laccase activity (U) in the filtrate after entrapment reaction (equivalent in µg) | Total free laccase activity (U) considering a 68% recovery after centrifuge ultrafiltration (equivalent in µg) | %EE = 100*(initial activity - total free activity)/initial activity | average %EE (standard deviation) |
| --- | --- | --- | --- | --- |
| 1.56 (13) | 0.94 (7.87) | 1.39 (11.6) | 11.12 | 41.9 (27.7) |
|  | 0.37 (3.12) | 0.55 (4.6) | 64.79 |  |
|  | 0.53 (4.43) | 0.78 (6.5) | 49.91 |  |
| 3.91 (33) | 1.97 (16.62) | 2.90 (24.4) | 25.85 | 29.5 (6.7) |
|  | 1.98 (16.7) | 2.91 (24.6) | 25.47 |  |
|  | 1.66 (14.04) | 2.45 (20.7) | 37.34 |  |
| 7.81 (66) | 3.21 (27.14) | 4.72 (39.9) | 39.54 | 24.3 (13.2) |
|  | 4.36 (36.88) | 6.42 (54.2) | 17.83 |  |
|  | 4.48 (37.86) | 6.59 (55.7) | 15.64 |  |
| 11.72 (99) | 4.69 (39.62) | 6.90 (58.3) | 41.13 | 51.8 (12.2) |
|  | 2.78 (23.48) | 4.09 (34.5) | 65.10 |  |
|  | 4.06 (34.3) | 5.97 (50.4) | 49.03 |  |
| 15.62 (132) | 10.24 (86.54) | 15.06 (127.3) | 3.59 | 8.9 (5.8) |
|  | 9.78 (82.65) | 14.38 (121.5) | 7.92 |  |
|  | 9.02 (71.62) | 13.26 (105.3) | 15.11 |  |

S2 table. **Data for calculating the % of entrapment efficiency (%EE)**. The %EE was calculated from activity values obtained for entrapment reactions and subsequent separation of free and entrapped enzyme. Initial units (and in parenthesis, expressed as µg) were calculated according to the volume of a stock of enzyme used in each entrapment reaction. The enzyme concentration and volumetric activity were 13.24 mg/mL and 1562 U/mL, respectively.

| Initial laccase activity in entrapment reaction (U) (equivalent in µg) | Total free laccase activity (U) considering a 68% recovery after centrifuge ultrafiltration (equivalent in µg) | Calculated U of entrapped laccase (equivalent in µg) | Amount of polymer produced in the presence of each amount of laccase (mg) (in parenthesis, concentration of polymer in mg/mL in the 600 µL reaction) | Laccase load in nanoparticles (µg laccase/g nanoparticles) | Average laccase load in nanoparticles, (in parenthesis is the standard deviation) (µg laccase/g nanoparticles) |
| --- | --- | --- | --- | --- | --- |
| 1.56 (13) | 1.39 (11.6) | 0.17 (1.4) | 11.45 (19.1) | 122.3 | 496 (372) |
|  | 0.55 (4.6) | 1.01 (8.4) |  | 733.6 |  |
|  | 0.78 (6.5) | 0.78 (6.5) |  | 567.7 |  |
| 3.91 (33) | 2.90 (24.4) | 1 (8.6) | 10.49 (17.5) | 819.8 | 931 (212) |
|  | 2.91 (24.6) | 1.01 (8.4) |  | 800.8 |  |
|  | 2.45 (20.7) | 1.46 (12.3) |  | 1172.5 |  |
| 7.81 (66) | 4.72 (39.9) | 3.09 (26.1) | 10.55 (17.6) | 2473.9 | 1524 (827) |
|  | 6.42 (54.2) | 1.39 (11.8) |  | 1118.5 |  |
|  | 6.59 (55.7) | 1.22 (10.3) |  | 976.3 |  |
| 11.72 (99) | 6.90 (58.3) | 5.63 (40.7) | 11.48 (19.1) | 3545.3 | 4468 (1054) |
|  | 4.09 (34.5) | 7.63 (64.5) |  | 5618.5 |  |
|  | 5.97 (50.4) | 5.75 (48.6) |  | 4233.4 |  |
| 15.62 (132) | 15.06 (127.3) | 0.56 (4.7) | 10.06 (16.7) | 467.2 | 1166 (764) |
|  | 14.38 (121.5) | 1.24 (10.5) |  | 1043.7 |  |
|  | 13.26 (105.3) | 2.36 (26.7) |  | 2654.1 |  |
| 0 | Not applicable | Not applicable | 14.4 (24.5) | Not applicable | Not applicable |

S3 table. **Data for calculating enzyme load in nanoparticles**. For each entrapment reaction, the entrapped enzyme was calculated as shown in the table, using the calculated entrapped activity and the experimentally measured amount of polymer produced in the 600 µL entrapment reaction.

Free laccase. No inactivation was detected after 1 h incubation at 25ᵒC

| **Residual activity after incubation at 25ᵒC** | U/ml | Average U/mL | Standard deviation |
| --- | --- | --- | --- |
| R1 | 1566.875 | 1562.167 | 7.100103 |
| R2 | 1554 |  |  |
| R3 | 1565.625 |  |  |

| Free laccase |  | **Residual activity after incubation at 40 ᵒC** | |  |  |  |  |  |
| --- | --- | --- | --- | --- | --- | --- | --- | --- |
|  | U/ml | | | % of activity | | | |  |
| Time (h) | R1 | R2 | R3 | R1 | R2 | R3 | Ave. of % of activity | Standard Deviation |
| 0 | 1.5125 | 1.357813 | 1.5625 | 100 | 100 | 100 | 100.00 | 0 |
| 10 | 1.235938 | 1.340625 | 1.5625 | 81.71 | 98.73 | 100.00 | 93.48 | 10.21114325 |
| 20 | 1.29375 | 1.471875 | 1.470313 | 85.54 | 108.40 | 94.10 | 96.01 | 11.55100287 |
| 30 | 1.307813 | 1.385938 | 1.396875 | 86.47 | 102.07 | 89.40 | 92.65 | 8.293202825 |
| 40 | 1.403125 | 1.245313 | 1.473438 | 92.77 | 91.71 | 94.30 | 92.93 | 1.300018866 |
| 50 | 1.2375 | 1.371875 | 1.398438 | 81.82 | 101.04 | 89.50 | 90.78 | 9.672935275 |
| 60 | 1.257813 | 1.4 | 1.35 | 83.16 | 103.11 | 86.40 | 90.89 | 10.7039871 |

| Free laccase |  | **Residual activity after incubation at 50 ᵒC** | |  |  |  |  |  |
| --- | --- | --- | --- | --- | --- | --- | --- | --- |
|  | U/ml | | | % of activity | | | |  |
| Time (h) | R1 | R2 | R3 | R1 | R2 | R3 | Ave. of % of activity | Standard Deviation |
| 0 | 1.478125 | 1.515625 | 1.442188 | 100 | 100 | 100 | 100.00 | 0 |
| 10 | 1.276563 | 1.40625 | 1.49375 | 86.36 | 92.78 | 103.58 | 94.24 | 8.697880979 |
| 20 | 1.510938 | 1.3125 | 1.440625 | 102.22 | 86.60 | 99.89 | 96.24 | 8.428013 |
| 30 | 1.31875 | 1.3625 | 1.476563 | 89.22 | 89.90 | 102.38 | 93.83 | 7.41299105 |
| 40 | 1.323438 | 1.496875 | 1.478125 | 89.53 | 98.76 | 102.49 | 96.93 | 6.670144826 |
| 50 | 1.240625 | 1.320313 | 1.242188 | 83.93 | 87.11 | 86.13 | 85.73 | 1.628965449 |
| 60 | 1.065625 | 1.278125 | 1.390625 | 72.09 | 84.33 | 96.42 | 84.28 | 12.16590853 |

| Free laccase |  | **Residual activity after incubation at 60 ᵒC** | |  |  |  |  |  |
| --- | --- | --- | --- | --- | --- | --- | --- | --- |
|  | U/ml | | | % of activity | | | |  |
| Time (h) | R1 | R2 | R3 | R1 | R2 | R3 | Ave. of % of activity | Standard Deviation |
| 0 | 0.931 | 0.942 | 0.905 | 100 | 100 | 100 | 100.00 | 0 |
| 10 | 0.845 | 0.851 | 0.543 | 90.76 | 90.34 | 60.00 | 80.37 | 17.63998896 |
| 20 | 0.786 | 0.722 | 0.711 | 84.43 | 76.65 | 78.56 | 79.88 | 4.053126844 |
| 30 | 0.676 | 0.758 | 0.798 | 72.61 | 80.47 | 88.18 | 80.42 | 7.783465591 |
| 40 | 0.716 | 0.79 | 0.704 | 76.91 | 83.86 | 77.79 | 79.52 | 3.787755774 |
| 50 | 0.706 | 0.79 | 0.671 | 75.83 | 83.86 | 74.14 | 77.95 | 5.193706085 |
| 60 | 0.706 | 0.735 | 0.647 | 75.83 | 78.03 | 71.49 | 75.12 | 3.325191857 |

| Free laccase |  | **Residual activity after incubation at 70 ᵒC** | |  |  |  |  |  |
| --- | --- | --- | --- | --- | --- | --- | --- | --- |
|  | U/ml | | | % of activity | | | |  |
| Time (h) | R1 | R2 | R3 | R1 | R2 | R3 | Ave. of % of activity | Standard Deviation |
| 0 | 1.526563 | 1.703125 | 1.259375 | 100 | 100 | 100 | 100.00 | 0 |
| 10 | 1.071875 | 0.778125 | 0.914063 | 70.21 | 45.69 | 72.58 | 62.83 | 14.89056931 |
| 20 | 0.43125 | 0.39375 | 0.573438 | 28.25 | 23.12 | 45.53 | 32.30 | 11.74342233 |
| 30 | 0.390625 | 0.379688 | 0.353125 | 25.59 | 22.29 | 28.04 | 25.31 | 2.883369231 |
| 40 | 0.176563 | 0.28125 | 0.25 | 11.57 | 16.51 | 19.85 | 15.98 | 4.168551965 |

| LNP-Lac 99 |  | **Residual activity after incubation at 70 ᵒC** | |  |  |  |  |  |
| --- | --- | --- | --- | --- | --- | --- | --- | --- |
|  | U/ml | | | % of activity | | | |  |
| Time (h) | R1 | R2 | R3 | R1 | R2 | R3 | Ave. of % of activity | Standard Deviation |
| 0 | 5.734375 | 5.375 | 12.51563 | 100 | 100 | 100 | 100.00 | 0 |
| 10 | 2.359375 | 1.875 | 1.804688 | 41.14 | 34.88 | 14.42 | 30.15 | 13.97738622 |
| 20 | 4.320313 | 5.312447 | 2.067688 | 75.34 | 98.84 | 16.52 | 63.57 | 42.40209781 |
| 30 | 2.976563 | 3.932252 | 0.534375 | 51.91 | 73.16 | 4.27 | 43.11 | 35.27646931 |
| 40 | 3.062469 | 2.734348 | 0.385156 | 53.41 | 50.87 | 3.08 | 35.78 | 28.35376994 |
| 50 | 2.286435 | 1.963522 | sample was finished | 39.87 | 36.53 |  | 38.20 | 2.363010643 |
| 60 | 2.18227 | 2.363281 | sample was finished | 38.06 | 43.97 |  | 41.01 | 4.180479903 |

S4 table. **Data for calculating thermal stability of LNP-Lac 99 µg.** Residual activity of free laccase was measured at various times upon incubation at several temperatures. Residual activity of LNP-Lac 99 μg was measured at various times upon incubation at 70 ᵒC

| Activity in U/mL | |  |  |  |  |  |  |  |  | |  |  |  |
| --- | --- | --- | --- | --- | --- | --- | --- | --- | --- | --- | --- | --- | --- |
| Storage time (days) | Repetition | LNP-Lac 66 µg | LNP-Lac 99 µg | LNP-Lac 132 µg | Repetition | LNP-Lac 66 µg | LNP-Lac 99 µg | LNP-Lac 132 µg | Repetition | | LNP-Lac 66 µg | LNP-Lac 99 µg | LNP-Lac 132 µg |
| 0 | R1 | 1.155 | 0.698 | 1.270 | R2 | 0.319 | 1.004 | 1.080 | R3 | | 0.239 | 0.704 | 0.921 |
| 6 |  | 0.580 | 0.645 | 1.227 |  | 0.282 | 0.757 | 0.807 |  | | 0.384 | 0.718 | 0.899 |
| 17 |  | 0.837 | 0.543 | 0.941 |  | 0.180 | 0.765 | 0.809 |  | | 0.271 | 0.699 | 1.278 |
| 27 |  | 0.738 | 0.766 | 1.520 |  | 0.213 | 0.820 | 1.460 |  | | 0.385 | 0.760 | 1.28 |
|  |  |  |  |  |  |  |  |  |  | |  |  |  |
| Activity as percentage of initial activity at time 0 | | | |  |  |  |  |  |  | |  |  |  |
| Storage time (days) | Repetition | LNP-Lac 66 µg | LNP-Lac 99 µg | LNP-Lac 132 µg | Repetition | LNP-Lac 66 µg | LNP-Lac 99 µg | LNP-Lac 132 µg | Repetition | | LNP-Lac 66 µg | LNP-Lac 99 µg | LNP-Lac 132 µg |
| 0 | R1 | 100.0 | 100.0 | 100.0 | R2 | 100.0 | 100.0 | 100.0 | R3 | | 100.0 | 100.0 | 100.0 |
| 6 |  | 50.2 | 92.4 | 96.6 |  | 88.4 | 75.4 | 74.7 |  | | 160.7 | 102.0 | 97.6 |
| 17 |  | 72.5 | 77.8 | 74.1 |  | 56.4 | 76.2 | 74.9 |  | | 113.4 | 99.3 | 138.8 |
| 27 |  | 63.9 | 109.7 | 119.7 |  | 66.8 | 81.7 | 135.2 |  | | 161.1 | 108.0 | 139.0 |
|  |  |  |  |  |  |  |  |  |  | |  |  |  |
| Average activity as percentage of initial activity at time 0 | | | | |  |  |  |  |  | |  |  |  |
| Storage time (days) | LNP-Lac 66 µg | LNP-Lac 99 µg | LNP-Lac 132 µg | Standard deviation for LNP-Lac 66 µg | Standard deviation for LNP-Lac 99 µg | Standard deviation for LNP-Lac 132 µg |  |  | |  |  |  |  |
| 0 | 100.0 | 100.0 | 100.0 | 0 | 0 | 0 |  |  |  | |  |  |  |
| 6 | 99.8 | 89.9 | 89.6 | 56.1 | 13.5 | 12.9 |  |  |  | |  |  |  |
| 17 | 80.8 | 84.4 | 95.9 | 29.4 | 12.9 | 37.1 |  |  |  | |  |  |  |
| 27 | 97.3 | 99.8 | 131.3 | 55.3 | 15.7 | 10.2 |  |  |  | |  |  |  |

S5 table. **Data for calculating storage stability of LNP-Lac preparations.** Initial rate was measured under standard conditions at different storage times, for samples kept at 4°C. Residual activity is expressed as percentage of the measured activity immediately after finishing the entrapment reaction and washing to eliminate free laccase.

|  | Free Laccase | | | |  |  |
| --- | --- | --- | --- | --- | --- | --- |
| Syringaldazine concentration (µM) | Initial rate in µM/min | | | |  |  |
|  | R1 | R2 | R3 | Average | Standard deviation | % error |
| 0 | 0.0 | 0.0 | 0.0 | 0.0 | 0.0 | 0.0 |
| 3 | 362.5 | 340.6 | 535.1 | 412.8 | 106.5 | 25.8 |
| 5 | 456.3 | 525.0 | 581.3 | 520.8 | 62.6 | 12.0 |
| 10 | 731.3 | 640.6 | 784.4 | 718.7 | 72.7 | 10.1 |
| 15 | 1015.6 | 743.8 | 1084.4 | 947.9 | 180.1 | 19.0 |
| 20 | 1065.6 | 1131.3 | 1175.0 | 1124.0 | 55.1 | 4.9 |
| 30 | 1465.6 | 1084.4 | 1000.0 | 1183.3 | 248.1 | 21.0 |
| 40 | 1237.5 | 1206.3 | 1168.8 | 1204.2 | 34.4 | 2.9 |
| 50 | 1100.0 | 1075.0 | 1087.5 | 1087.5 | 12.5 | 1.1 |
| 60 | 1231.3 | 1175.0 | 1187.0 | 1197.8 | 29.6 | 2.5 |

|  | LNP-Lac 99 µg | | | |  |  |
| --- | --- | --- | --- | --- | --- | --- |
| Syringaldazine concentration (µM) | Initial rate in µM/min | | | |  |  |
|  | R1 | R2 | R3 | Average | Standard deviation | % error |
| 0 | 0.0 | 0.0 | 0.0 | 0.0 | 0.0 | 0.0 |
| 3 | 1.32 | 1.29 | 1.42 | 1.34 | 0.07 | 5.2 |
| 5 | 1.78 | 1.66 | 1.69 | 1.71 | 0.07 | 3.9 |
| 10 | 2.80 | 2.64 | 2.83 | 2.75 | 0.10 | 3.7 |
| 15 | 3.80 | 3.84 | 3.95 | 3.86 | 0.08 | 2.1 |
| 20 | 4.45 | 4.13 | 4.31 | 4.30 | 0.16 | 3.8 |
| 30 | 6.20 | 4.64 | 5.39 | 5.41 | 0.78 | 14.4 |
| 40 | 5.69 | 5.77 | 6.84 | 6.10 | 0.65 | 10.6 |
| 50 | 7.22 | 6.20 | 4.63 | 6.02 | 1.30 | 21.7 |
| 60 | 7.65 | 5.84 | 4.86 | 6.12 | 1.42 | 23.1 |

S6 table. **Data for performing the non-linear regression to obtain the catalytic parameters Vmax and Km.** The values are referred to stock preparations, considering all dilutions**.** Non-linear regression was performed using Prism 8. kcat was calculated from the obtained value of Vmax considering Vmax=kcat [enzyme concentration]. For the free enzyme, the concentration of the stock was 13.24 mg/mL, and purity of 90% and a molecular weight of 61,000 Da were considered. For LNP-Lac 99 µg, a concentration of entrapped enzyme of 0.308 mg/mL (calculated from 51% EE and a sample volume of 0.167 mL, and considering 7.5 µL of the stock at 13.24 mg/mL were used for this entrapment reaction), as well as a purity of 90% and a molecular weight of 61,000 Da, were considered.
